# Low-Density Lipoprotein Receptor LRP-2 regulates GLR-1 glutamate receptors and glutamatergic behavior in *C. elegans*

**DOI:** 10.17912/micropub.biology.000837

**Published:** 2023-04-26

**Authors:** Bethany J Rennich, Eric S Luth, Julia Hofer, Peter Juo

**Affiliations:** 1 Program in Neuroscience, Graduate School of Biomedical Sciences, Tufts University School of Medicine, Boston, MA 02111; 2 Developmental, Molecular and Chemical Biology, Tufts University School of Medicine, Boston, MA 02111; 3 Biology, Simmons University, Boston, MA 02115

## Abstract

We identified the Low-Density Lipoprotein (LDL) Receptor Related Protein-2 (LRP-2) in a RNAi screen for genes that regulate glutamatergic behavior in *C. elegans* . *lrp-2* loss-of-function mutants have defects in glutamatergic mechanosensory nose-touch behavior and suppress increased spontaneous reversals induced by GLR-1(A/T), a constitutively-active form of the AMPA-type glutamate receptor GLR-1. Total and surface levels of GLR-1 are increased throughout the ventral nerve cord of *lrp-2* mutants suggesting that LRP-2 promotes glutamatergic signaling by regulating some aspect of GLR-1 trafficking, localization or function.

**Figure 1. LPR-2 regulates GLR-1 and glutamatergic behavior:**
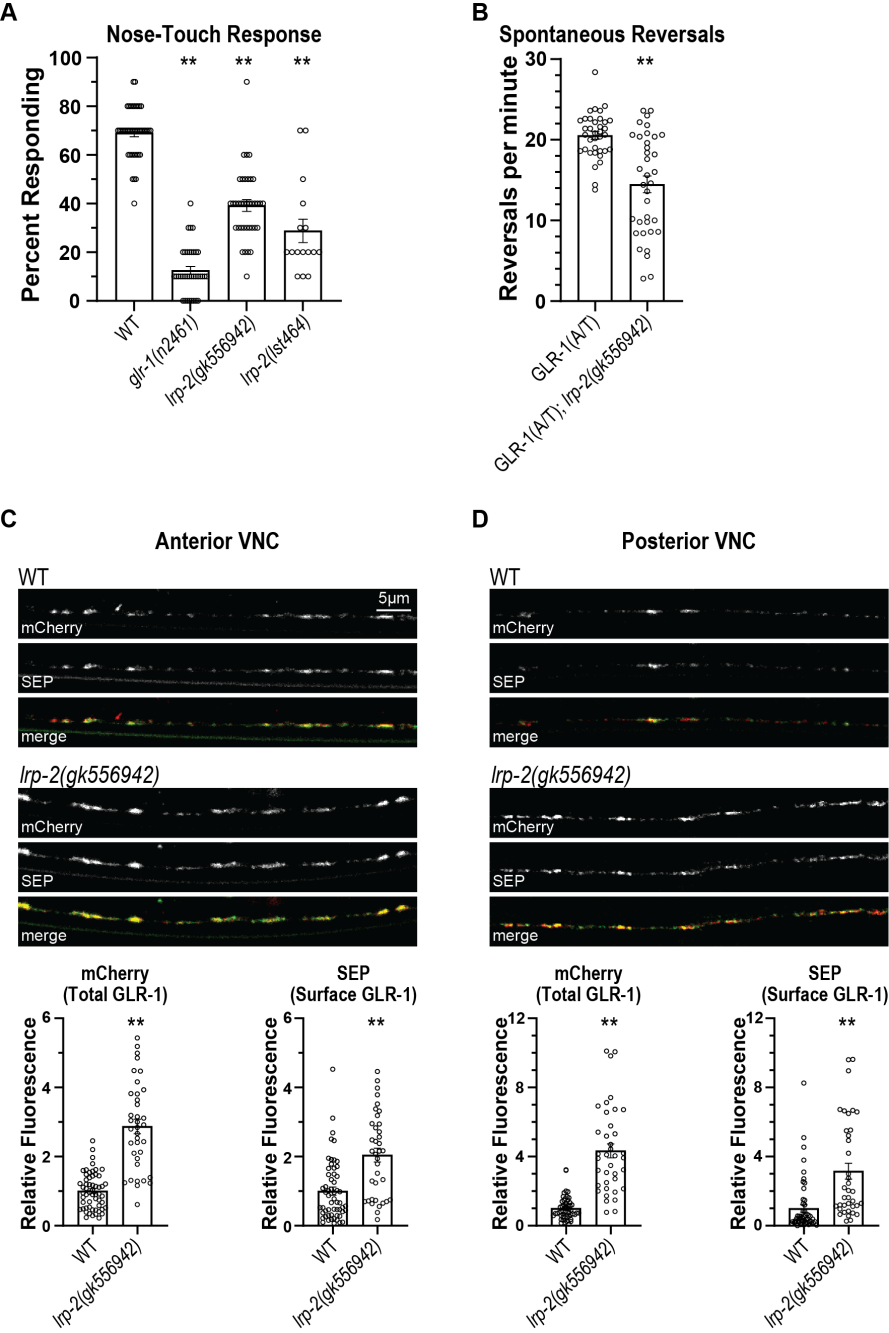
(A) Response to nose-touch in WT, *glr-1(n2461)* , *lrp-2(gk556942)* (6x backcrossed) and *lrp-2(lst464)* (2x backcrossed) (n=16-37 per genotype). Percent Responding (Mean +/- SEM) is shown. One-way ANOVA with Sidak’s Multiple Comparison Test: **p<0.0001 vs WT. (B) Spontaneous locomotion reversal frequencies are shown for animals expressing constitutively-active GLR-1, GLR-1(A/T) ( *nuIs80* ), in the absence or presence of *lrp-2(gk556942)* . Average Reversals/min +/-SEM are shown (n=36). Student’s *t* test: **p<0.0001. (C-D) Representative images of SEP::mCherry::GLR-1 ( *akIs201* ) in the anterior (C) and posterior (D) VNC of AVA in WT and *lrp-2(gk556942)* mutants. Quantification of mCherry (total GLR-1) and SEP (surface GLR-1) fluorescence in the indicated genotypes are shown below the images. (n=37-57). Average fluorescence (Norm.) +/- SEM are graphed. Student’s *t* test: **p<0.0001.

## Description

We performed a RNAi screen for genes that regulate glutamatergic behavior as previously described (Luth et al., 2021) . Mechanical stimulation of the nose of the worm with an eyelash activates a locomotion reversal behavior called the nose-touch response (Hart et al., 1995; Kaplan and Horvitz, 1993; Maricq et al., 1995) . A pair of glutamatergic sensory neurons ASH, as well as other neurons, detect this nose-touch resulting in activation of GLR-1 AMPA-type glutamate receptors (AMPARs) on downstream interneurons such as AVA (Hart et al., 1995; Kaplan and Horvitz, 1993; Maricq et al., 1995; Mellem et al., 2002; Piggott et al., 2011) . Expression of Channelrhodopsin specifically in ASH enables optogenetic activation of ASH, which results in glutamate-dependent calcium transients and depolarization of AVA interneurons and locomotion reversals (Ezcurra et al., 2011; Guo et al., 2009; Lindsay et al., 2011) . We used this optogenetic ASH (optoASH) assay to photostimulate animals after RNAi knockdown of genes with cell adhesion molecule domains and monitored their reversal responses as described (Luth et al., 2021) . We identified the Low-Density Lipoprotein (LDL) Receptor-Related Protein-2 (LRP-2) in this screen.

We validated our *lrp-2* RNAi findings with two independent loss of function alleles of *lrp-2* , *lrp-2(gk556942)* and *lrp-2(lst464)* . Both *lrp-2(gk556942)* and *lrp-2(lst464)* exhibit the “bag of worms” phenotype and are likely null mutations (Van Rompay et al., 2015) . Both *lrp-2* mutants contain non-sense mutations: *gk556942* contains a R750Stop and *lst464* contains a C3875Stop (Van Rompay et al., 2015) . We found that *lrp-2(gk556942)* (6 times backcrossed) and *lrp-2(lst464)* (2 times backcrossed) are nose-touch defective using the traditional eyelash-induced nose-touch assay ( Figure 1A ), consistent with a defect in glutamatergic behavior. We found that *lrp-2(gk556942)* mutants have wild-type rates of thrashing (Average Thrashes/minute (+/-SEM): N2: 62.2+7.3 (n=24); *lrp-2(gk556942)* : 67.6+8.2 (n=24), p>0.05, n.s., Student’s *t* test), suggesting that *lrp-2* mutants do not have a general defect in neuromuscular junction function.

We next tested if loss of *lrp-2* alters spontaneous locomotion reversals, another glutamatergic behavior. Spontaneous reversal frequency is mediated by glutamate and GLR-1 (Zheng et al., 1999) . Mutants lacking glutamate release, such as worms lacking the presynaptic vesicular glutamate transporter *eat-4* /VGLUT or postsynaptic *glr-1* , or mutants with decreased synaptic GLR-1 or function (Brockie et al., 2001; Burbea et al., 2002; Kowalski et al., 2011; Mellem et al., 2002; Zhang et al., 2012; Zheng et al., 2004) exhibit reduced frequencies of spontaneous reversals. In contrast, worms expressing a constitutively-active form of GLR-1 (GLR-1(A/T)) (Zheng et al., 1999) or mutants with increased levels of GLR-1 at synapses (Juo and Kaplan, 2004; Schaefer and Rongo, 2006) exhibit increased rates of spontaneous reversals. Mutants with defects in synaptic GLR-1 levels or trafficking (Kowalski et al., 2011; Luth et al., 2021) or GLR-1 function (Zheng et al., 2004) can suppress the increased spontaneous reversals observed in GLR-1(A/T) expressing animals. We found that *lrp-2(gk556942)* mutants suppress the increased reversal frequency induced by GLR-1(A/T) ( Figure 1B ). Because the effects of GLR-1(A/T) on GLR-1 signaling are independent of presynaptic glutamate release (Zheng et al., 1999) , this data is consistent with a postsynaptic site of action for *lrp-2* , perhaps to regulate GLR-1 levels, distribution or function.

We analyzed the levels and distribution of GLR-1 in the ventral nerve cord (VNC) using a transgenic strain expressing a functional GFP-tagged GLR-1 ( *nuIs24* : *glr-1p::GLR-1::GFP* ) (Rongo et al., 1998) in *lrp-2* mutants. We found that the peak intensity and size of GLR-1::GFP puncta were increased in the posterior VNC (GLR-1::GFP Average peak puncta fluorescence intensity ± SEM (A.U.): WT: 419.92 ± 51.35, *lrp-2(gk556942)* : 633.04 ± 70.46, p<0.0001, Student’s *t* test; GLR-1::GFP Average puncta width ± SEM (µm): WT: 1.38 ± 0.11, *lrp-2(gk556942)* : 2.15 ± 0.16, p<0.01, Student’s *t* test), suggesting that GLR-1 levels and distribution are disrupted in *lrp-2* mutants. Previous studies have found that increased accumulation of GLR-1::GFP levels in the VNC can be correlated with increased (Juo and Kaplan, 2004; Schaefer and Rongo, 2006) or decreased (Glodowski et al., 2007; Rongo et al., 1998) glutamatergic signaling because measurements of GFP-tagged GLR-1 cannot distinguish between surface and internal pools of the receptor. Thus, we directly measured total and surface levels of GLR-1 in AVA neurons using a functional GLR-1 tagged on its extracellular N-terminus with SuperEcliptic pHluorin (SEP) and mCherry ( *rig-3p::SEP::mCherry::GLR-1* ), as previously described (Hoerndli et al., 2013; Hoerndli et al., 2015) . SEP is a pH-sensitive fluorophore and only fluoresces in the neutral pH of the extracellular space. SEP fluorescence is quenched in the acidic environment of endosomes and thus SEP fluorescence represents GLR-1 at the neuronal surface. In contrast, mCherry fluoresces at the cell surface and inside endosomes and thus mCherry fluorescence represents total GLR-1 levels. Consistent with our results with GLR-1::GFP, we found that *lrp-2(gk556942)* mutants had increased total (mCherry fluorescence) levels of GLR-1 in the anterior ( Figure 1C ) and posterior ( Figure 1D ) VNC. However, surprisingly, we found that surface levels (SEP fluorescence) of GLR-1 were also increased throughout the VNC of *lrp-2* mutants ( Figure 1C -D).

LRP-2 is homologous to LRP1 in mammals (Willnow et al., 2012) . LRP1 is well-known as a multifunctional endocytic receptor that promotes the internalization of over 40 diverse ligands including LDL, factor VIII, extracellular matrix proteins, viruses and Alzheimer’s Precursor Protein (APP) (Herz and Strickland, 2001; Kounnas et al., 1995; Liu et al., 2000) . LRP1 also has non-endocytic roles and regulates intracellular signaling and morphogen trafficking. Several functions for *lrp-2* in *C. elegans* have been described including the regulation of EGL-17/FGF secretion (Kamikura and Cooper, 2003) , vulva cell precursor lineage polarity (Minor and Sternberg, 2019) and yolk protein synthesis (Van Rompay et al., 2015) .

Our data suggest that *lrp-2* is required for glutamatergic signaling in *C. elegans* . We found that *lrp-2* mutants have defects in glutamatergic nose-touch behavior and suppress the increased spontaneous reversals induced by constitutively-active GLR-1(A/T). In mammals, LRP1 is highly expressed in the nervous system and localizes to the postsynaptic density (Nakajima et al., 2013) . LRP1 can regulate AMPAR signaling and synaptic plasticity (Gan et al., 2014; Liu et al., 2010; Martin et al., 2008) , however there are conflicting reports regarding its role in long-term potentiation (LTP) (May et al., 2004) . Liu et al. (2010) reported that LRP1 is required for LTP in the hippocampus based on the conditional knockout of LRP1 in excitatory forebrain neurons in mice (Liu et al., 2010) . In contrast, May et al. (2004) found no change in hippocampal LTP in a conditional knockout of LRP1 using synapsin-Cre mice (May et al., 2004) . Another study found that LRP1 mediates the enhancement of LTP by Tissue-type Plasminogen Activator (tPA) (Zhuo et al., 2000) . In this case the effect of LRP1 is likely mediated by direct interaction with the scaffold PSD95 and NMDA receptors (NMDARs) and regulation of NMDAR-mediated calcium signaling (Bacskai et al., 2000; Martin et al., 2008; May et al., 2004) . LRP1 also interacts with AMPARs and controls their trafficking, however the role of LRP1 in regulating AMPAR trafficking is also controversial (Gan et al., 2014; Nakajima et al., 2013) . Gan et al. (2014) showed that RNAi knockdown of LRP1 in cortical neurons leads to decreased total and surface levels of the AMPAR subunit GluA1 via increased internalization and degradation (Gan et al., 2014) . In contrast, Nakajima et al. (2013) found that there was no change in surface or total levels of GluA1 under basal conditions in cortical neurons cultured from LRP1 knock-out mice. However, NMDA-induced internalization of GluA1 was dependent on LRP1 (Nakajima et al., 2013) , suggesting that LRP1 is required for the endocytosis of AMPARs in response to NMDA treatment.

We found that total and surface levels of GLR-1 increase in vivo in *lrp-2* mutants throughout the VNC in *C. elegans* . Given that LRP-2 promotes the internalization of a large number of proteins, our data might be consistent with a role for LRP-2 in promoting GLR-1 endocytosis. However, the fact that *lrp-2* mutants also have defects in GLR-1-dependent behaviors suggests that the pool of GLR-1 that accumulates at the neuronal surface in *lrp-2* mutants is not functional. Mammalian LRP1 can promote calcium signaling via GluA1 homomers (Gan et al., 2014) and NMDARs in cultured rodent neurons (Martin et al., 2008) . Given that GLR-1 is permeable to calcium (Zheng et al., 1999) and LRP1 interacts with mammalian AMPARs (Gan et al., 2014) , it is possible that LRP-2 may directly promote GLR-1 function in *C. elegans* . The increase in total GLR-1 observed in *lrp-2* mutants could be due to a compensatory upregulation of GLR-1 expression, as we have previously shown happens in response to decreased glutamatergic signaling (Moss et al., 2016) . We speculate that LRP-2 acts via more than one mechanism to regulate GLR-1 function, localization and/or trafficking.

## Methods

**RNAi screen**

*lrp-2* was found in a RNAi screen described in (Luth et al., 2021) . Briefly, genes with cell adhesion molecule domains were knocked down using feeding RNAi in a strain enhanced for neuronal RNAi (FJ1300) and were screened in triplicate for defective backward movement in response to optogenetic activation of ASH sensory neurons as previously described (Luth et al., 2021) .

**Nose-touch assay**

The nose touch assay was carried out on NGM plates that were spotted with OP50 (diluted 1:10 with LB broth) the day before the experiment and dried overnight. The experimenter was blinded to all genotypes. Individual animals were picked to the assay plate, then an eyelash attached to the end of a wooden stick was placed in the path of the worm. A trial was counted if the nose of the worm collided perpendicularly with the eyelash. A trial was scored as a reversal if the worm immediately initiated a backward movement a distance greater than the length of the nose to the posterior pharyngeal bulb. Ten trials were completed for each worm assayed.

**Thrashing**

Thrashing was performed as described previously (Luth et al., 2021) . The experimenter was blinded to all genotypes. Briefly, individual worms were picked to a 5 µL drop of M9 Buffer on a glass coverslip and allowed to acclimate for ~1 minute. Following the acclimation period, a timer was set for 1 minute and the number of thrashes was counted using a handheld tally device. A thrash was defined as the worm moving through a C shape curled ventrally to dorsally and back.

**Spontaneous reversal assay**

NGM Assay plates were poured the day before the experiment and dried overnight. The experimenter was blinded to all genotypes. Individual animals were picked with halocarbon oil to the unspotted assay plate and were allowed to acclimate for 2 minutes. The number of spontaneous reversals in a 5-minute period were counted and recorded. Reversals were defined as backward movement greater than the distance from the nose to the posterior bulb of the pharynx.

**Epifluorescence imaging and quantification**

Imaging of GLR-1::GFP in *nuIs24* was carried out on a Carl Zeiss Axiovert M1 microscope with a 100x Plan Apochromat objective (1.4 numerical aperture) as previously described (Luth et al., 2021) . An 11 plane Z-stack with 1 µm total thickness captured the posterior VNC. A maximum intensity projection of the Z-stack was used to generate a linescan using Metamorph software (v7.1, Molecular Devices). Linescans were analyzed using custom-written software (IgorPro v6, Wavemetrics) (Burbea 2002, Kowalski 2011) . Puncta were identified using a minimum peak width of 0.3 µm and a minimum peak threshold 4 standard deviations above the VNC background fluorescence. Puncta fluorescence intensities were normalized to the mean intensity of standardized fluorescent beads for each corresponding day of imaging. Puncta width was defined as the average of the widths of each punctum at half maximal peak intensity.

**Confocal imaging and quantification**

The VNC of worms expressing SEP::mCherry::GLR-1 ( *akIs201* ) was imaged using a Zeiss LSM800 confocal microscope with a 63X Plan Apochromat objective (numerical aperture 1.4). Identical acquisition settings were used for all images: 101.51 µm X 58.68 µm image size; z-stack: 18 slices (6.63 µm total thickness), 488 nm laser: 0.4% with 776V gain, 561 nm laser: 0.3% with 867V gain. Quantification of mCherry and SEP fluorescence was performed using a custom MATLAB script. The script was fed an average background fluorescence for the mCherry and SEP channels set from representative data sets, respectively, from wild type animals to threshold all images. The script facilitates drawing a ROI around the VNC that is applied to both the mCherry and SEP channels and used to measure total fluorescence within the ROI. For each imaging session, experimental genotypes were normalized to wild type so imaging sessions could be aggregated. Representative images were processed equivalently in ImageJ for display.

## Reagents

**Table:** 

Genotyping primers for:	Forward primer	Reverse primer
*lrp-2(gk556942)*	5’ ACAGTTCACGGACTTACTGG 3’	5’ CACATGCATTCGGATCCACC 3’
*lrp-2(lst464)*	5’ AGGAATGCGCAATTGTTTGT 3’	5’ CCTGCCGATCAAACAAGACT 3’

**Table:** 

Strain	Genotype	Source
N2	Wild type	CGC
AQ2235	*lite-1(ce314) ljIs114* ( *gpa-13p::FLPase, sra-6p::FTP::ChR2::YFP* ) X	William Schafer
TU340	*sid-1(pk3321);uIs69(unc-119p::sid-1;myo-2p::mCherry) V*	Martin Chalfie
FJ1300	*lin-35(n745) nuIs25 I; sid-1(pk3321) uIs69 V; lite-1 (ce314) ljIs114 X*	Luth et al. (2021)
FJ1672	*lrp-2(gk556942) (6x backcrossed) I*	CGC and this study
LSC904	*lrp-2(lst464) (2x backcrossed) I*	Liesbet Temmerman
KP1147	*nuIs24 (glr-1p::GLR-1::GFP) IV*	Joshua M. Kaplan
FJ1596	*nuIs24 IV; lrp-2(gk556942) I*	This study
KP2006	*nuIs80 (glr-1p::GLR-1(A/T)::YFP)*	Joshua M. Kaplan
FJ1662	*nuIs80; lrp-2(gk556942) I*	This study
FJ1499	*akIs201 (rig-3p::SEP::mCherry::GLR-1) V*	Andres V. Maricq
FJ1659	*akIs201 V; lrp-2(gk556942) I*	This study
